# Manipulation of spermatogonial stem cells in livestock species

**DOI:** 10.1186/s40104-019-0355-4

**Published:** 2019-06-12

**Authors:** Filipp Savvulidi, Martin Ptacek, Karina Savvulidi Vargova, Ludek Stadnik

**Affiliations:** 1 Department of Animal Science, Faculty of Agrobiology, Food and Natural Resources, Czech University of Life Sciences Prague, Kamýcká 129, 165 00 Prague, Suchdol Czech Republic; 2 Institute of Pathological Physiology, First Faculty of Medicine, Charles University in Prague, U Nemocnice 5, 128 53 Prague, Czech Republic

**Keywords:** CRISPR/Cas9, Genome editing, Livestock transgenesis, Long-term culture systems, Male germline stem cells, Recipient preparation, Sertoli cells, Spermatogonial stem cells, Ultrasonographic-guided cannulation

## Abstract

We are entering an exciting epoch in livestock biotechnology during which the fundamental approaches (such as transgenesis, spermatozoa cryopreservation and artificial insemination) will be enhanced based on the modern understanding of the biology of spermatogonial stem cells (SSCs) combined with the outstanding recent advances in genomic editing technologies and *in vitro* cell culture systems. The general aim of this review is to outline comprehensively the promising applications of SSC manipulation that could in the nearest future find practical application in livestock breeding. Here, we will focus on 1) the basics of mammalian SSC biology; 2) the approaches for SSC isolation and purification; 3) the available *in vitro* systems for the stable expansion of isolated SSCs; 4) a discussion of how the manipulation of SSCs can accelerate livestock transgenesis; 5) a thorough overview of the techniques of SSC transplantation in livestock species (including the preparation of recipients for SSC transplantation, the ultrasonographic-guided SSC transplantation technique in large farm animals, and the perspectives to improve further the SSC transplantation efficiency), and finally, 6) why SSC transplantation is valuable to extend the techniques of spermatozoa cryopreservation and/or artificial insemination. For situations where no reliable data have yet been obtained for a particular livestock species, we will rely on the data obtained from studies conducted in rodents because the knowledge gained from rodent research is translatable to livestock species to a great extent. On the other hand, we will draw special attention to situations where such translation is not possible.

**Electronic supplementary material:**

The online version of this article (10.1186/s40104-019-0355-4) contains supplementary material, which is available to authorized users.

## Introduction to mammalian spermatogenesis

### Spermatogonial stem cells

Spermatogenesis is a delicately orchestrated process of continuous sperm cell production when billions of haploid spermatozoa are produced daily. The reproductive system of sexually mature males requires the presence of a stem cell pool to maintain an extremely high level of daily sperm production (as high as 31.3 billion of spermatozoa in a boar [1], 12.9 billion in a ram [2], 11.5 billion in a bull [3], 8 billion in a stallion [4] or 6.4 billion in a buck [5], [reviewed in [6]).

The process of mammalian spermatogenesis occurs in the seminiferous tubules, the gametogenic compartment of the mammalian testes, and in the interstitium, where the steroidogenic activity in the testis occurs. SSCs, a group of spermatogonia, represent the foundation of spermatogenesis. These cells have a unique self-renewal ability (the ability to divide without differentiation) and a differentiation ability while giving rise to the haploid spermatozoa [7]. SSCs originate from the gonocytes (also known as prespermatogonia) in the postnatal testes. Gonocytes, in turn, originate from the primordial germ cells (PGCs) during fetal development [8–11]. Collectively, PGCs, gonocytes and SSCs are all referred to as male germline stem cells (MGSCs) because of their stem cell potential [12]. Among these, the SSCs are the overall focus of this review.

### SSC self-renewal: vital support from the niche

In postnatal mammalian testes, SSCs reside within a specialized microenvironmental compartment of the seminiferous epithelium located near the basement membrane. These compartments, populated by SSCs and Sertoli cells, are canonically called niches. Moreover, there are several niche-related cell types and structures in the testicular interstitium (Leydig cells, the interstitial macrophages, interstitial lymphatic endothelial [LE] cells and vasculature) and in the seminiferous peritubular region (peritubular myoid cells [PMCs] and peritubular macrophages), which act via paracrine signaling to mutually regulate the SSC fate by balancing SSC self-renewal and differentiation in the niche [13].

The processes of niche organization, SSC self-renewal and differentiation, and spermatogonial development in mammals is complex and is under the control of the myriad of paracrine molecular factors. Readers interested in these processes should refer to the excellent reviews published elsewhere: [13–19], and very recently, [20]. However, for the purpose of the present review, it is important to briefly outline the niche-related cells and their main molecular factors, which play a role in the processes of niche organization and, especially, in the maintenance of SSC self-renewal.

Sertoli cells (the only cells in the niche that directly contact germ cells) produce several molecular factors, which are essential for SSC self-renewal. Among these, we will focus on the glial cell line-derived neurotrophic factor (GDNF) and the basic fibroblast growth factor (bFGF). From GDNF knockout studies (discussed in [13]), it is known that GDNF signaling is crucial for SSC self-renewal *in vivo*. Therefore, GDNF is the most common molecular growth factor used in SSC culture to maintain self-renewal *in vitro*. bFGF appears to play a minor supportive role in SSC self-renewal because this factor alone cannot fully support the self-renewal of murine SSCs [21]. Interestingly, *in vitro* cocultivation experiments showed that the secretion of both GDNF and bFGF is upregulated in Sertoli cells in response to the withdrawal of SSCs from the culture [22]. *in vivo*, this mode of action could stimulate self-renewal and abrogate the differentiation of SSCs.

For the purposes of present review, it is important to mention the critical role the Sertoli cells plays in the formation of the so-called blood–testes barrier. This physical (although not absolute) barrier, formed by the Sertoli cell - Sertoli cell tight junctions, separates the spermatogonia in the basal compartment of the seminiferous epithelium from the spermatocytes and spermatids in the apical compartment [23].

Leydig cells produce colony-stimulating factor 1 (CSF1) and insulin-like growth factor 1 (IGF1); both of these factors increase the proliferation of SSCs; however, the mitogenic effects of both molecules are only achieved in concert with GDNF [24, 25]. Leydig cells also stimulate gonad development and maintain spermatogenesis via testosterone production [26].

The role of testicular macrophages within the SSC niche is discussed. Macrophages produce 25-hydroxycholesterol, an intermediate compound within the testosterone biosynthetic pathway; therefore, macrophages might speed up testosterone production in the testes [27, 28]. DeFalco et al. [29] proposed that macrophages may contribute to the proliferation/self-renewal through the secretion of CSF1.

Testicular vasculature also plays an important role in maintenance of SSC self-renewal. It has been observed in bulls [30] and in mice [31] that the vascular endothelial growth factor (VEGF) positively regulates germ cell proliferation. DeFalco et al. reported that CSF1 is released by the perivascular smooth muscle cells [29].

Very recently, it was reported that the subset of interstitial lymphatic endothelial (LE) cells near the vasculature secrete fibroblast growth factor family ligands that act as SSC mitogens [20].

Although limited attention has been paid to the role of PMCs in the process of SSC self-renewal, Chen et al. showed in their study in mice that testosterone induces the secretion of GDNF by PMCs *in vitro* and that the production of GDNF by PMCs is crucial for undifferentiated spermatogonia development and maintenance *in vivo* [32]. However, it is not the only influence that PMCs have on SSCs: the expression of CSF1 in PMCs was also reported [25, 29].

**Fig. 1:**
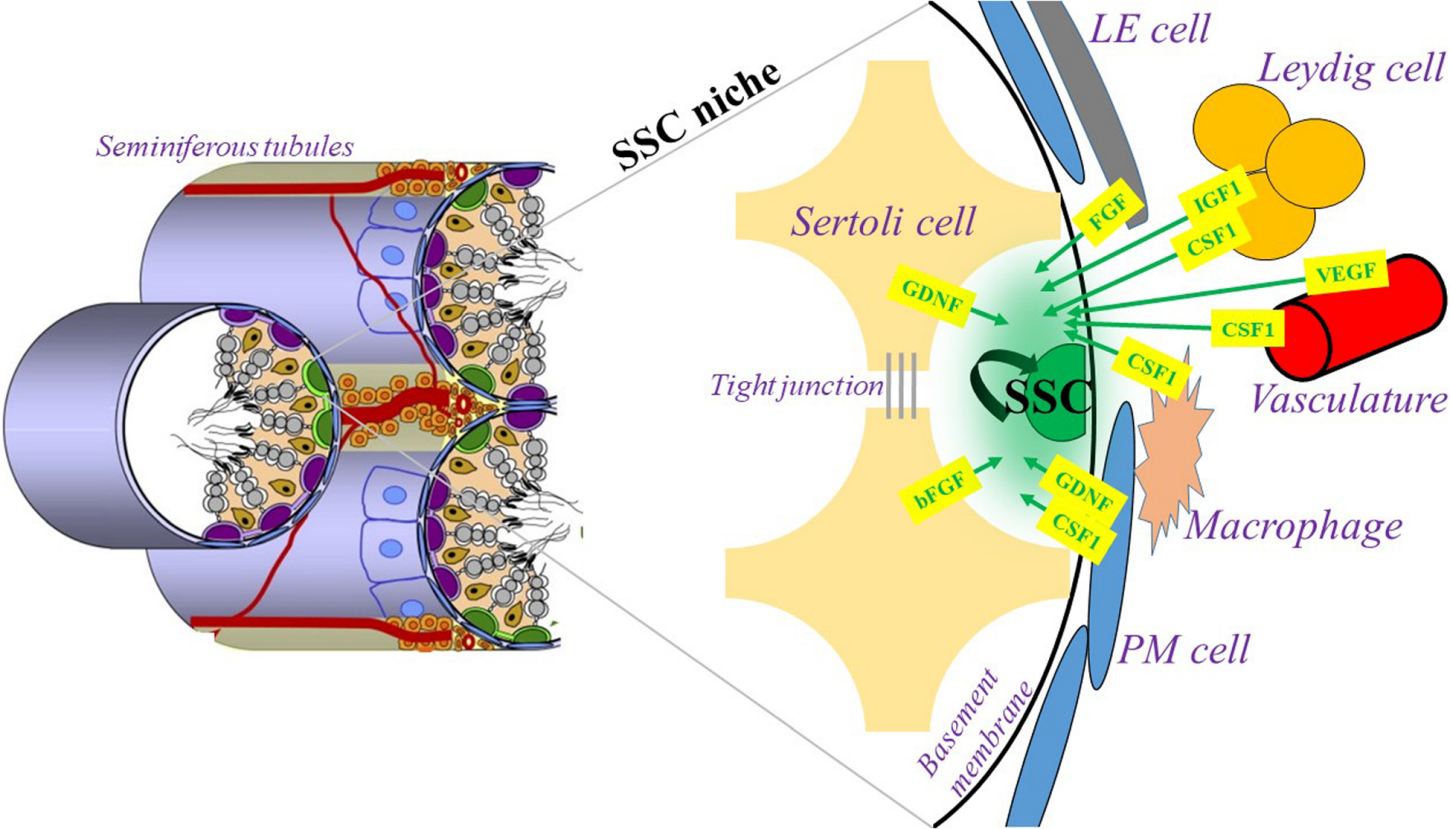
Schematic representation of the SSC niche. GDNF - glial cell line-derived neurotropic factor. bFGF - basic fibroblast growth factor. CSF1 - colony-stimulating factor 1. VEGF - vascular endothelial growth factor. IGF1 – insulin-like growth factor 1. LE cell – interstitial lymphatic endothelial cells. PM cell - peritubular myoid cells. SSC - spermatogonial stem cell with the self-renewal ability. Adapted from [184], modified

A schematic representation of the SSC niche is given in Fig. 1.

## Approaches for SSC isolation, purification and expansion of isolated SSCs *in vitro*

### SSC harvesting from the testes of donor animals

In the testes of adult animals, the SSCs represent a very rare cell population (0.01% - 0.03%) [33, 34]. Therefore, neonatal or prepubertal testes are the preferred source for harvesting SSCs because spermatogonia are the major cell type present in the seminiferous tubules during those developmental stages [35]. Certain interventions such as surgical induction of cryptorchidism, vitamin A deficiency or hyperthermic treatment (to eliminate differentiating germ cells [36, 37]) or polythiouracil-induced hypothyroidism (to increase the number of Sertoli cells [38]) might result in the *in vivo* enrichment of SSC in the testes of adult animals. However, the rate of enrichment varies between livestock species (discussed in [39]).

In general, before SSC purification, the interstitial tissue from the seminiferous tubules is dissociated by collagenase and hyaluronidase digestion. Afterward, the seminiferous tubules are incubated with trypsin-EDTA to release the SSCs and to obtain a single-cell suspension; DNase (an enzyme that catalyzes the degradation of DNA) is added to prevent cellular aggregation [35]. This single-cell suspension of testicular cells is composed of SSCs and somatic cells.

### SSC purification approaches: Immunostaining

To purify the SSCs from the somatic cells, different approaches might be used. FACS (fluorescence-activated cell sorting) or MACS (magnetic activated cell sorting) immunostaining approaches are based on the recognition of the molecular phenotype of the SSCs. The PGP9.5 (protein gene product 9.5) molecular marker is expressed in premeiotic male germ cells and does not show an affinity for somatic cells, which makes it an optimal marker to recognize spermatogonia in several livestock species [40–42]. However, a limited number of previously discovered and canonically used phenotype-specific molecular markers to identify and isolate SSCs from livestock species (especially in species other than bull and boar) is heavily obstructing progress in the field. Therefore, the search for novel phenotype-specific molecular markers for ovine, caprine or equine SSCs is of great importance. For ovine, CDH1 (also known as E-cadherin) is a novel molecular marker that can be used together with PLZF (the promyelocytic leukemia zinc finger protein), the other well-known marker to identify undifferentiated spermatogonia [43]. Interestingly, to identify the SSCs among the isolated testicular cells in birds, immunostaining for several phenotype-specific mammalian molecular markers might be used. Pramod et al. identified the SSC subset of spermatogonial cells in Japanese Quail by immunostaining the testicular cells with the DAZL (deleted in azoospermia-like), ITGA6 (integrin alpha-6) and GFRα1 (GDNF family receptor alpha-1) molecular markers, which are routinely used for SSC identification in mammals [44].

**Fig. 2:**
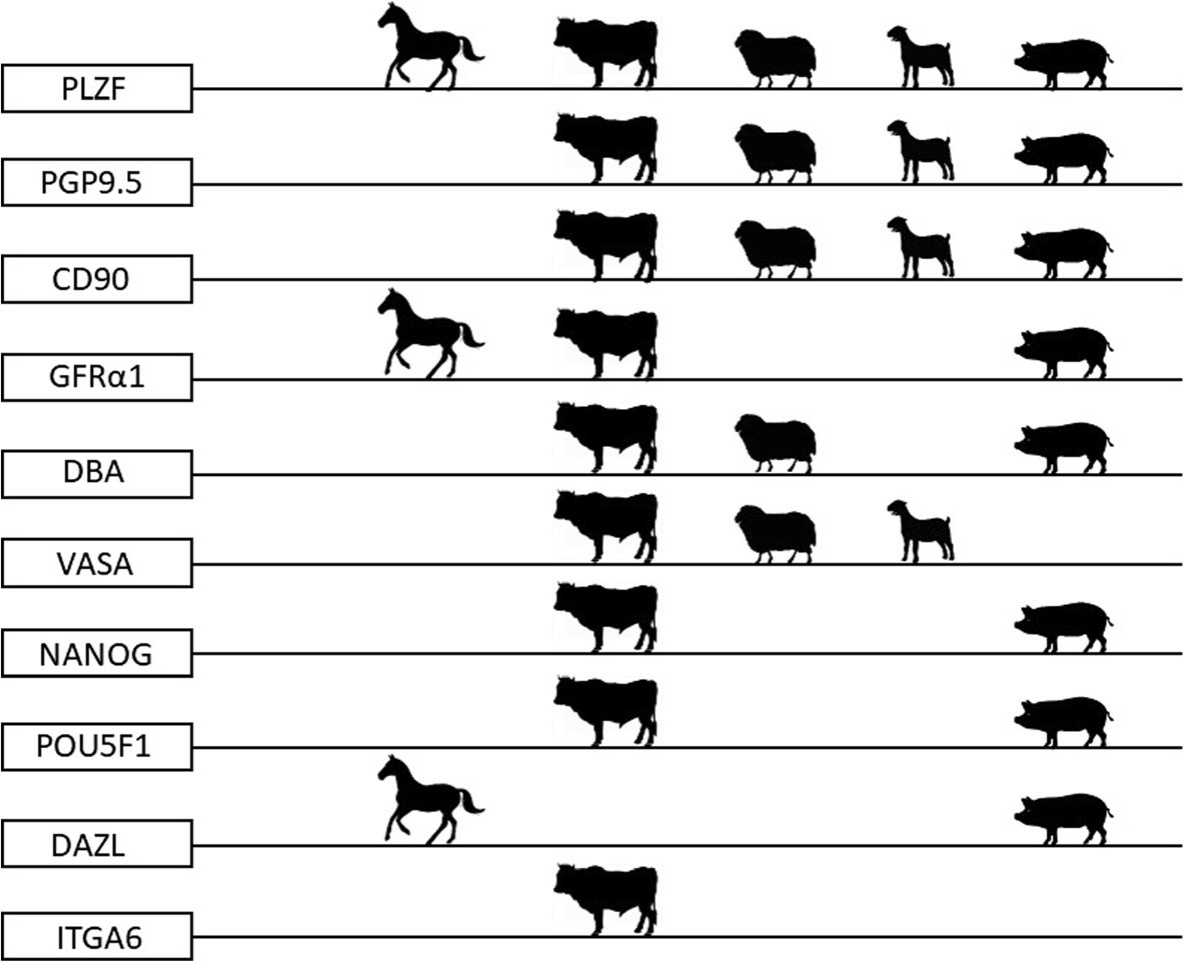
Phenotype-specific molecular markers to identify and isolate SSCs from several livestock species. PLZF (also known as ZBTB16) - promyelocytic leukemia zinc-finger protein, a transcription factor essential for the maintenance and self-renewal of SSC. PGP9.5 (protein gen product 9.5; also known as UCHL1) – ubiquitin carboxyl-terminal hydrolase L1, is expressed in a premeiotic male germ cells and does not show an affinity for somatic cells, which makes it an optimal marker for spermatogonia in domestic testes. CD90 (also known as THY1, thymocyte differentiating antigen) – claster of differentiation 90. GFRα1 - GDNF family receptor α1. DBA - lectin *Dolichos biflorus* agglutinin. VASA (also known as DEAD-box polypeptide 4, DDX4). NANOG, transcription factor related to the pluripotency of stem cells. POU5F1 (also known as Oct3/4) – POU domain, class 5, a transcription factor related to the pluripotency of stem cells. DAZL - deleted in azoospermialike, a protein localized in the nucleus of spermatogonia. Similar to several other molecular markers presented in the figure, an expression of this protein is stage-dependent. DAZL protein relocates to the cytoplasm during meiosis where it persists in spermatids and spermatozoa. ITGA6 - Integrin Subunit Alpha 6, protein, mammalian SSC molecular marker. Readers interested in the original reference for each phenotype-specific molecular marker should refer to the excellent reviews published elsewhere [26, 135, 185]

In relation to FACS, it is worth mentioning that the purification of spermatogonia based solely on their light scatter properties was proven to be successful in salmonid fishes [45] and recently in boars [46]. In the latter study, a simple approach to isolate spermatogonia using FACS was established by the authors. With this approach, the population of spermatogonia was sorted based on their forward and side light scatter [46]. No knowledge of the surface proteins presents on spermatogonia or the availability of highly selective antibodies was required for this approach. Furthermore, the sorting of cell populations based on the light scatter properties does not require the staining of cells with nucleic acid dyes such as Hoechst 33342. This dye labels the cell nuclei with blue fluorescence and is a canonical probe for flow cytometry of sperm and somatic cells. An important drawback of Hoechst is that the flow cytometer must be equipped with an ultraviolet laser, which can substantially increase the cost of the machine [47] (Fig. 2).

#### Other approaches for SSC purification

Apart from the phenotype-based approaches (FACS, MACS), other approaches such as Staput velocity sedimentation (the separation of SSCs through a linear bovine serum albumin (BSA) gradient) and differential plating (the selection of SSCs with the use of extracellular matrix components) equally serves as common practice for SSC enrichment.

Using the Staput apparatus, the fraction enriched for SSCs can be collected out of the initially heterogeneous population of testicular cells [48, 49]. This is because the individual types of testicular cell sediments in the BSA gradient have different sedimentation velocities according to cell size and mass. In contrast to most FACS or MACS protocols, the Staput method does not require DNA or any other types of staining. An advantage of the Staput method over FACS is the ability to isolate highly viable SSCs suitable for subsequent culture after Staput [48]. The Staput method was successfully applied for the enrichment of SSCs from buck testes with an average yield of 6 × 10^8^ PGP9.5 positive and highly viable SSCs out of an initial heterogeneous population of 10^9^ testicular cells [49].

In the differential plating technique, gelatin, lectin and laminin can be used for the selection of SSCs. Somatic cells (Sertoli cells, Leydig cells, myoid and peritubular cells) have an affinity towards gelatin and lectin, thus leaving SSCs in the supernatant when differentially plated. The α- and β-integrin receptors of SSCs efficiently bind to the laminin, resulting in an enrichment of SSCs through positive selection [50]. Using these approaches, SSCs have been enriched to as high as 75% in the population of donor testis cells from bull, ram or boar, summarized by Honaramooz and Yang [51].

Importantly, all of the above-mentioned approaches are mainly suitable for isolating SSC from large volumes of testicular tissue and, therefore, could fit only experimental settings. Because the castration or surgical removal of a part of a testis is not usually possible in an elite animal, testicular biopsy is considered instead [39]. Germline stem cells (including SSCs) represent a rare cell population in testes and the number of germline stem cells is a limiting factor for achieving success in the eventual cell genome editing and/or cell transplantation [12]. Therefore, the ability to expand male germline stem cells [Additional file 1] *in vitro* is crucial.

### The expansion of livestock MGSC *in vitro*

Culture conditions for male germline stem cell expansion *in vitro* were first established in rodents; these conditions and concepts behind this expansion are also the basis for the development of male germline stem cell culture systems in livestock species [52]. Soon after GDNF was identified as a major molecular factor required for SSC self-renewal *in vivo* [53], a short-term culture system supplemented with GDNF was developed for male mouse germline stem cells *in vitro* [54]. Long-term cultures of SSCs were achieved by adding several other growth factors and hormones in addition to the recognized GDNF [55].

However, the direct transfer of culture conditions established in rodents to livestock species is unfortunately not possible [26]. For instance, although gonocytes and SSCs isolated from neonatal and immature bull testes can be maintained in culture conditions established for mice, the SSCs from adult bull testes can be maintained in long-term culture only in the presence of 6-bromoindirubin-3′-oxime, an inhibitor of glycogen synthase kinase-3α, which is not crucial for maintaining mouse SSCs [56]. These results indicate that bull and mouse germ cells need different factors for growth. Interestingly, it is known that the inhibition of glycogen synthase kinase-3α leads to the activation of the Wnt/β-catenin signaling pathway. As recently reported in tree shrews, the Wnt/β-catenin signaling pathway is involved in the maintenance of undifferentiated spermatogonia isolated from adult testes during the early stages of *in vitro* culture [57].

Since the very first attempts to establish a culture system for SSCs, serum has been used as an important component in the culture medium for supporting the survival and self-renewal of cultured cells [11]. However, it is known that some undefined factors in serum induce cell differentiation, and other factors might have detrimental effects on germ-cell survival in the culture [58]. To overcome this problem, serum-free culture systems have been developed for SSCs in rodents [58–61] and in non-rodents [11, 56, 62]. In addition, the utilization of feeder-free culture systems may be more efficacious for expanding spermatogonia *in vitro* in comparison to the feeder-based systems because feeders present a variable component that is not possible to standardize [11]. On the other hand, a Sertoli cell feeder was shown to support the survival and the *in vitro* expansion of SSCs in rat [63], Japanese quail [44] and tree shrew [57].

It is known that the proliferation of cultured SSCs decreases over time, and differentiation and apoptosis dominate over the propagation of undifferentiated SSCs [64]. Oxidative stress and apoptosis are the most common injuries to SSCs [65]. Therefore, it might be a valuable approach to include antioxidants in SSC culture to prevent the accumulation of toxic products from the metabolism and the formation of reactive oxygen species. For instance, in a recent publication [66], the impact of two antioxidants, vitamin C and Trolox, alone or in combination, on the SSC medium of bull calves was evaluated. The obtained results demonstrated that vitamin C and Trolox could improve SSCs viability in culture if added individually, but not simultaneously. These results warrant future studies to establish culture systems for germline stem cell expansion without the induction of apoptosis.

At present, an effective long-term cultures were successfully established in rabbit [67], bull [11, 56, 68], boar [62], buck [69], Japanese quail [44] and in tree shrew [57].

## Livestock transgenesis via manipulation of SSCs

### The approach of the genetic modification of livestock through the transplantation of genetically altered SSCs

The most promising application of SSC manipulation is the generation of transgenic farm animals, defined as the genetic modification of livestock through the transplantation of genetically altered SSCs for improving productivity and commercial value.

Historically, the first genetically modified livestock, including rabbits, sheep, and pigs, were produced by pronuclear microinjection (PM, the injection of foreign DNA into the pronucleus of a fertilized oocyte) [70]. Next, somatic cell nuclear transfer (SCNT or cloning), which involves the enucleation of mature oocytes, followed by the injection or fusion of the donor cell and the activation of the reconstructed embryo, has been used to generate transgenic goats [71], pigs [72], sheep [73], and cattle [74]. Unfortunately, both PM and SCNT are technically challenging, costly, time-consuming and inefficient [75]. In animals produced by PM, the exogenous transgene is usually inserted randomly into the genome, resulting in so-called target allele mosaicism. This phenomenon reflects independent gene editing events during early embryonic cleavage stages [76]. To generate colonies of nonmosaic germline mutants that are isogenic for the targeted alleles in all germ and somatic cells in their bodies, the target allele heterogeneity must be outcrossed [77]. The outcrossing of allelic mosaicism is relatively short in rodents but can require years in some livestock species due to longer life cycles and/or low fecundity [78]. This, in addition to its low efficiency (success rates are not above 10%), has prevented the use of PM on a large scale in livestock [75]. SCNT is the preferred approach for generating genetically modified large animals; however, somatic cells have a lower frequency of homology recombination in comparison to embryonic stem (ES) cells and have a limited life span in culture. This hampers the establishment of cell lines with the desired genetic modification. Furthermore, SCNT frequently induces developmental abnormalities in animals because of incomplete nuclear reprogramming [75].

The self-renewal and pluripotent characteristics of embryonic stem (ES) cells could provide advantages for livestock genetic modification by providing an opportunity for the longstanding screening of correctly modified cells or by improving the efficiency of cloning by nuclear transfer [79]. However, for large farm animals (except the recent success in bovine [80] [additional file 2] where authors were able to establish putative stable pluripotent bovine ES cells), no stable ES cell lines were established.

Therefore, the genetic modification of livestock currently relies on the approach utilizing the fascinating ability of *in vitro* genetically modified germ cells to colonize the recipient testes and to produce donor-derived sperm upon transplantation. The direct genetic modification of donor germ cells avoids the totipotent state of embryogenesis, thus eliminating the production of mosaic mutant progeny [81]. Furthermore, spermatogenesis *in vivo* provides a perfect natural selection to eliminate transgenic sperm with undesired mutations: when any genetic mutation is introduced that is incompatible with the survival, proliferation, or differentiation of SSCs, those SSCs will fail to form functional transgenic sperm, thus preventing the spread of undesired or lethal mutations [49]. Importantly, the time required for the production of genetically modified sperm is significantly shorter using germ cell transplantation compared to cloning or ES cell-based technology [75]. Thus, the approach of the genetic modification of livestock through the transplantation of genetically altered SSCs is currently being considered to complement the PM or SCNT for the production of transgenic farm animals [82] or genetically-edited birds [83].

### Genome editing in livestock MGSCs

In a broad spectrum of genome editing research the rodent models still represent a “gold standard”, mainly due to their perfect laboratory manageability (such as the small body size of adult animals, low cost of maintenance, short reproductive cycle and short life span). Importantly, the knowledge we learn from rodent research is translatable onto other species to a large extent: the essential concepts or ideas, first originated in rodent research, sooner or later become an integral part of the research in livestock or humans. For example, the concept of genomic editing in germline stem cells using engineered nucleases (an artificial genome editing reagents) first originated in mouse and rat research [81, 84–86] provided a platform for engineered nuclease-mediated genome editing in germline stem cells in large farm animals [46].

Generally, livestock MGSCs can be genetically edited either via the random integration of the exogenous transgene into the cell genome or by the approach of precise genome editing via engineered nucleases.

#### Genome editing via random integration of the exogenous transgene

As the proliferation of MGSCs *in vitro* is known to be slow, the prevailing method of MGSC genome editing through random integration commonly relies on the use of viral vectors [75]. The feasibility of viral vectors for this purpose was demonstrated in the very first report of livestock transgenesis via germline stem cell transplantation [87]. In that study, the authors used the adeno-associated virus (AAV) vector to insert an exogenous gene for green fluorescence protein (GFP) into the genome of buck germ cells. AAV is a small virus from the parvovirus family that is the preferred viral vector due to its ability to enter both proliferating and nonproliferating cells and (for *in vivo* experiments) due to its associated low risk of inducing host immune responses [88]. The next essential AAV advantage, is that AAV remains primarily episomal after entering into cell, so the random integration of the viral genetic material into the host genome is avoided [89].

There are several other types of viral vectors available (for example, based on lenti-, adeno-, or flaviviruses), and each type provides a unique set of advantages and limitations. The pros and cons of these types have already been extensively reviewed elsewhere [88, 90]. However, it is important to mention lentivirus (LV)-based vectors, which can also be used as an effective tool for the genomic editing of livestock MGSCs [82]. In this sense, an interesting study was published not long ago, where mice transgenesis was achieved by the lentiviral transduction of MGSCs *in vivo* [91]. In that study, a lentiviral vector containing a GFP transgene was injected into the mouse testes, resulting in the integration of the transgene into the host (pre-founder) genome at the injection site. Moreover, after the pre-founder males were mated with wild-type females of the same strain, the transgene was found to be expressed in 67.88% of the F1 offspring. This simple and efficient approach, if appropriately modified, could be applied to livestock species, leading to the advancement of livestock transgenesis.

#### Genome editing via engineered nucleases (aka precise genome editing)

The approach of precise genome editing is based on the use of three classes of engineered nucleases: zinc-finger nucleases (ZFN), transcription activator-like effector nucleases (TALEN) or RNA-guided nucleases (such as CRISPR/Cas9) [discussed in 84] to induce double-stranded breaks (DSB) in the DNA at very precise locations to initiate the nonhomologous end-joining (NHEJ) or homologous recombination (HR) repairing processes [additional file 3].

ZFN was the first engineered nuclease described for the approach of precise genome editing [92]. ZFN was demonstrated to edit the genome in mouse germline stem cells [84]. However, to the best of our knowledge, ZFN in livestock species was only successfully used for somatic cell genome editing in bulls [93] and boars (discussed in [94]); its use for the genomic editing of livestock MGSCs is limited, and the detailed discussion of ZFN is beyond the scope of the present review. Readers interested in genome editing via ZFN should refer to review published elsewhere [95].

TALEN, the second generation of engineered nucleases, has a similar structure to ZFN, consisting of a DNA recognition domain and *Fok*I endonuclease; however, their DNA recognition domains are different [96]. The TALEN DNA binding domain originates from the bacterial plant pathogen *Xanthomonas*, which is used by this bacterium to alter the expression of several host genes [97]. The TALEN engineered nuclease has a much simpler design, assembly, and a broader targeting range [98]. Furthermore, in comparison to ZFN, the smaller size of TALEN results in less steric hindrance and toxicity [99]. TALEN was used for genomic editing in chicken primordial germ cells, generating ovalbumin gene knockout chickens [100], or for targeting the *DDX4* locus and producing *DDX4* null offspring [101]. In a recent study, Tang et al. [46] reported the TALEN-mediated editing of the porcine Duchenne muscular dystrophy locus in boar spermatogonia. These reports are the first to confirm the achievability of precise genomic editing in livestock germ cells. However, commercially available TALENs are expensive, take weeks to obtain, and the technologies of protein engineering to produce TALENs do not guarantee active nucleases from a given design [102]. Fortunately, an engineered nuclease is available today which is superior to TALEN; compared to TALEN, this superior engineered nuclease is simpler, easier to construct, has a lower cytotoxicity and a higher targeting efficiency. We are now speaking about CRISPR/Cas9.

CRISPR (clustered regularly interspaced short palindromic repeats), together with its associated Cas9 nuclease, assembles into the CRISPR/Cas9 complex, which represents the most advanced generation of engineered nucleases today. Initially, CRISPR was identified as the adaptive immune system of bacteria against exogenous viral DNA or plasmid DNA [103].

In year 2012 Jinek et al. [104] reported the ability of a ribonucleoprotein complex of dual RNA (the so-called guide RNA; gRNA) consisting of a 20-base pair CRISPR RNA (crRNA) and the trans-activating crRNA (tracrRNA) together with the *Streptococcus pyogenes* type II Cas9 protein nuclease to induce DSB on DNA at very specific target sites. Since that report, the CRISPR/Cas9 system has been rapidly accepted as being fundamental for relatively simple, but precise, time- and cost-effective genome editing technology. The first application of the CRISPR/Cas9 system to edit the mammalian genome was reported in 2013 [105]. Compared to TALEN, CRISPR/Cas9 (due to its small size, which is only 20 base pairs) is simpler and easier to construct [106]. Furthermore, CRISPR/Cas9 has a lower cytotoxicity and a higher targeting efficiency [107]. Both the RNA and the protein components of the CRISPR/Cas9 ribonucleoprotein complex can be delivered into the cells in several different ways. The Cas9 protein can be delivered as a DNA expression plasmid, as an *in vitro* transcript, or as a recombinant protein bound to the RNA portion. The RNA component can be delivered either expressed as a DNA plasmid or as an *in vitro* transcript. There are pros and cons for each delivery method, primarily regarding the final CRISPR/Cas9 efficiency; these were thoroughly discussed elsewhere [102, 108]. Importantly, not only the NHEJ pathway but also the HR pathway could be induced by the CRISPR/Cas9 system; therefore, not only indels but also an exogenous DNA sequences could be introduced into a specific target site with the knock-in strategy [109, 110].

Moreover, multiplexed genome editing ability (the ability to target simultaneously two or more genes in a single cell) was demonstrated for the CRISPR/Cas9 system [105, 111], [112]. Multiple guiding sequences can be encoded into a single CRISPR array, which enables the simultaneous targeting of several sites within the genome. This multiplexed ability of the CRISPR/Cas9 system was extremely suitable for inactivating all 62 copies of porcine endogenous retrovirus (PERV) sequences in the genome of an immortalized porcine cell line [111]. Of great importance, with the use of CRISPR/Cas9 multiplexed genome editing in primary porcine fibroblasts, PERV-free pigs were generated through SCNT and embryo transfer [112].

Apart from studies in rodents [105, 81, 84–86], the feasibility of the CRISPR/Cas9 system for non-rodent MGSC genome editing was reported in chicken PGCs to target the chicken immunoglobulin heavy chain loci [113] or the ovomucoid egg white allergen gene [114]. In tree shrews (an animal, closely related to primates), CRISPR/Cas9 was also reported to be feasible for SSC genome editing; with the use of engineered CRIPSR/Cas9, the gene encoding amyloid β precursor protein *(App)* was successfully disrupted [57].

On the other hand, it is important to mention that the CRISPR/Cas9 system is not absolute: there is a concern for the off-target cleavage activity of CRISPR/Cas9 because the system only requires a recognition of 20 base pairs [104, 105] and allows up to five base pair mismatches for the formation of a DSB [115]. Consequently, if a normal, nontarget gene has enough homology to the target gene, it might also be inactivated by CRISPR/Cas9. To overcome its off-target issue, either the binding specificity of CRISPR/Cas9 could be improved based on the bioinformatic analysis or the implementation of a mutant “nickase” variants of Cas9 (Cas9-D10A or Cas9n) could be considered (discussed in [116]). In addition to the Cas9 endonuclease, a class 2-type V CRISPR effector nuclease called Cpf1 (CRISPR from *Prevotella* and *Francisella* 1) was recently identified [117]. This nuclease has higher accuracy and therefore has fewer off-target cleavage activities than Cas9 [118, 119]. These novel CRISPR variants with enhanced accuracy will further facilitate its broad applications in livestock MGSC genome editing.

## SSC transplantation: fundamental for the manipulation of spermatogonial stem cells in livestock species

### Preparation of the recipients for SSC transplantation

Unlike in rodent research, for SSC transplantation in livestock species, most researchers use immature recipients, and this is not exclusively due to the evident size-related reasons. The immature intratesticular microenvironment is more favorable than adult testes for the engraftment and expansion of donor SSCs [120].

An interesting phenomenon of immunotolerance to the transplanted genetically unrelated donor-derived SSCs has been observed in livestock species. Recipient pigs [121, 122], goats [123, 124], sheep [125, 126], and bulls [127] with fully functional immune systems did not reject germ cells from unrelated donors (discussed in [128]). The immune privilege of recipient testes was considered to explain the observed phenomenon. That is in contrast to rodents, where similar transplantation experiments resulted in limited colonization of recipient testes unless immune suppression was used (discussed in [128]). Although the reasons for this interspecies difference are not clear, it is generally accepted, that Sertoli cells play a critical role in granting the immunologic privilege to the testes. Given the limited contribution of the blood-testes barrier into the immunoprotection of germ cells [129, 130], it has been suggested and then confirmed in the *in vitro* studies and *in vivo* studies (where Sertoli cells survive transplantation across barrier) that Sertoli cells are potent immunomodulators and secrete a broad scale of immunosuppressive molecular factors, which are inhibitors of immune response. Among these, immunomodulating cytokines (transforming growth factor β, interleukin 6); inhibitors of B and T cells; inhibitors of complement system (decay-accelerating factor, DAF); inhibitors of granzime-, and FAS-FAS ligand-mediated apoptosis (protease inhibitor - 9) and anti-inflammatory prostanoids (prostaglandin E synthase) should be mentioned. Detailed overview of these factors is given elsewhere [128, 131–133]).

Again, unlike rodents, in livestock animal models, the elimination of the recipient’s endogenous SSCs to produce vacant niches for donor-derived SSCs is not a critical prerequisite [additional file 4], but it is a valuable approach for improving the result of SSC transplantation [51, 134, 135]. Indisputable, if SSC transplantation is used as a breeding tool, the elimination of the endogenous germline cells must be complete; otherwise, a mix of donor- and recipient-origin sperm will be produced after transplantation [136].

#### Elimination of the recipient’s endogenous SSC by busulfan treatment

The partial ablation of the recipient’s endogenous SSCs (the so-called gonadal ablation) can be achieved by the use of busulfan (1,4 - butanediol dimethanesulfonate), a chemotherapeutic drug [51]. Busulfan is an alkylating agent that induces apoptosis in dividing cells. Because SSCs are mitotically active, these cells are sensitive to mitosis inhibition [137] and to apoptosis induced by busulfan [136]. While busulfan treatment may appear practical in field conditions (as no expensive equipment is required), it has a clear disadvantage related to its systemic toxicity. The dose-dependent species-specific systemic toxicity of busulfan during treatment was demonstrated in pigs [138] and lambs [139]; therefore, researchers must be aware of the busulfan-related toxicity danger. In lambs, a dose of 8 mg/kg induced thin diarrhea with lethargy and a lack of appetite beginning at 5 days post busulfan injection; the authors suggested using reduced doses of busulfan [139]. Because of the systemic toxicity of busulfan treatment in postnatal pigs, in utero busulfan treatment (i.e., the administration of busulfan to sows in their late gestation to coincide with the period of male germ cell proliferation in the fetus) can be utilized instead [138].

Nevertheless, the huge disadvantage of busulfan is its systemic toxicity (i.e., the possibility of damaging all organs or systems in the animals after busulfan treatment). Moreover, busulfan does produce a biohazard concern, as it is a chemotherapeutic drug and is eliminated from the treated animals via the feces and urine [136].

Busulfan treatment not only eliminates the endogenous SSCs but also damages the Sertoli cells [140, 141].

Taken together, busulfan treatment must be considered after all other methods of recipient endogenous SSC elimination have been considered.

#### Elimination of the recipient’s endogenous SSCs by local testicular irradiation

The irradiation efficiency depends strongly on the irradiation dose and animal age at time of irradiation, and dramatic interspecies differences in response to irradiation in livestock were observed [39]. Although testicular irradiation is the preferred method for the ablation of endogenous germ cells in large animals [126, 135, 136] and does not cause a biohazard concern as a treatment regimen in livestock [136], it can still negatively affect the neighboring somatic support cells. Relatively high irradiation doses (> 5–6 Gy) which are required to eliminate endogenous spermatogonia in rodents, non-rodents and avian species were shown to compromise the viability of Sertoli cells and caused testicular atrophy [139]. Moderate irradiation doses, although not killing the germ cells, were shown to result in the block of spermatogonial differentiation due to injury to the neighboring somatic compartment in rats [142]. In very young lambs, the Sertoli cells can regenerate, and their numbers return to normal after irradiation; however, their supporting functionality is evidently corrupted [139, 142]. In young bulls, it was shown that testicular irradiation (at dose of 10 Gy) can damage the function of the Leydig cells, which can impair testosterone secretion throughout adulthood [136]. This is a serious disadvantage of the irradiation approach; the restoration of spermatogenesis following irradiation and SSC transplantation can be challenging due to impaired testosterone secretion. Moreover, some endogenous spermatogonia might still survive after serious damage by irradiation and, if developing into spermatids, might transferring gained genomic aberrations to the next generation [143]. Another disadvantage of the procedure of testicular irradiation is the requirement in a large-sized, sophisticated and expensive radiation source and the requirement of anesthesia to perform the treatment appropriately [135]. Therefore, the availability of the testicular irradiation procedure is limited in field conditions.

**Fig. 3:**
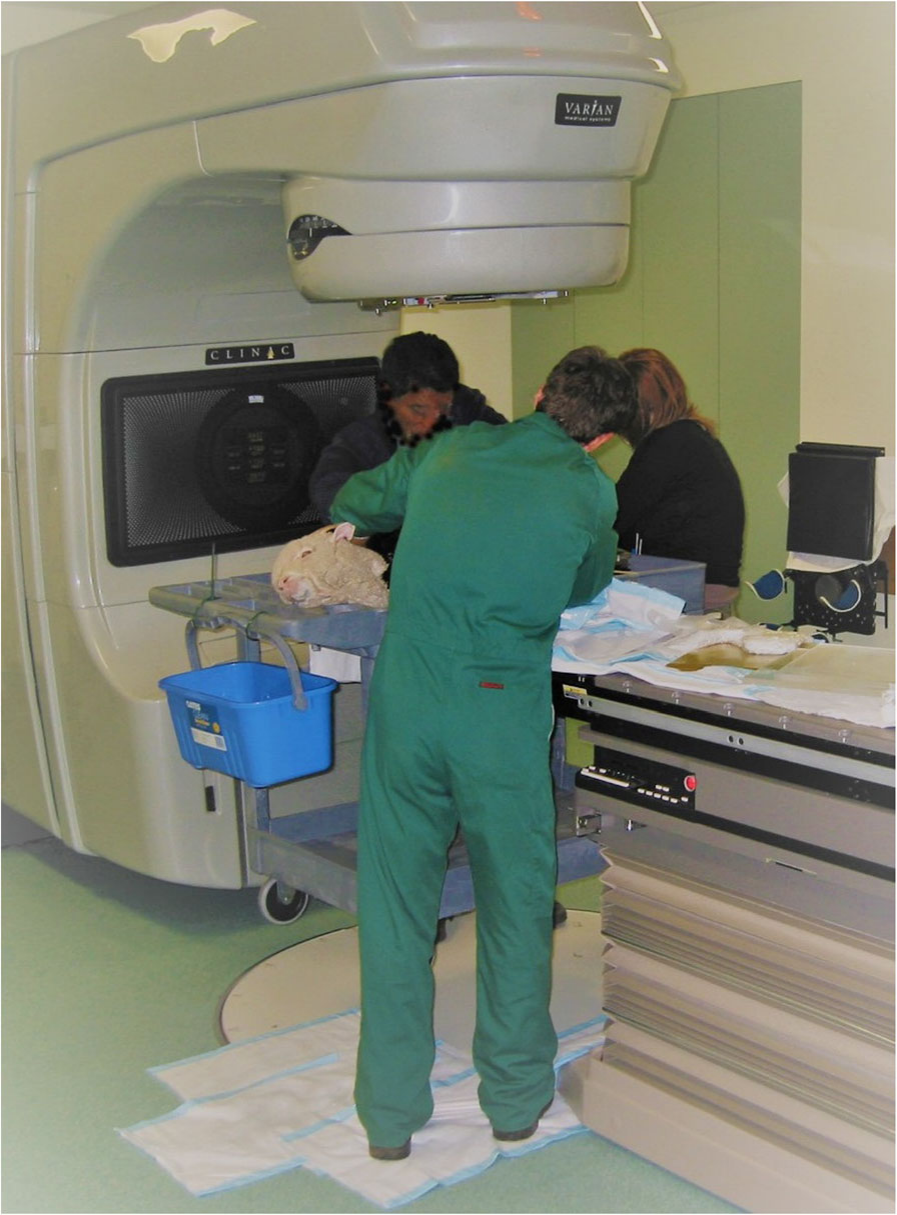
The procedure of testicular irradiation in ram. The radiation source in this image is produced by a linear accelerator which delivered a 6 MV photon beam directly to the testis at a dose rate of 2 Gy per minute. Reproduced with a permission from the personal archive of professor Jonathan Hill, PhD

Hence, the field is desperately requiring new techniques to improve the SSC transplantation efficiency or a better alternative to both the busulfan and testicular irradiation techniques (Fig. 3).

### Improvements in the SSC transplantation efficiency

Successful SSC transplantation can only be performed in recipients lacking their endogenous SSCs with preserved intact niches for the donor-derived SSCs. As discussed in the preceding chapters, gonadal ablation (busulfan treatment or testicular irradiation) can compromise the viability and functionality of Sertoli cells and the steroidogenic activity of Leydig cells. Therefore, the approach of the cotransplantation of non-compromised donor-derived niche components (mainly Sertoli and/or Leydig cells) together with donor-derived SSCs into gonadal-ablated recipients is of great interest [additional file 5].

#### Sertoli/SSCs cotransplantation

In mice, the ability of intratesticularly transplanted donor-derived Sertoli cells to form seminiferous tubule structures in the testes of infertile recipients with busulfan-compromised or genetically defective endogenous Sertoli cells was reported [144]. This is a proof of concept study, as it confirms the possibility of transplanting niche components (namely, Sertoli cells). Importantly, the morphogenic activity of Sertoli cells was shown to be enhanced if perinatal donor Sertoli cells were used for transplantation [144]. This is in concordance with the generally accepted suggestion that immature (prepubertal) Sertoli cells are able to proliferate [145], whereas mature Sertoli cells are mitotically quiescent [146]. Indeed, immature Sertoli cells derived from prepubertal cashmere buck testes were used to establish long-term *in vitro* culture with significant (up to 20 passages) proliferative potential and stable expression of germ cell regulatory factors [147]. Menegazzo et al. reported immature Sertoli cells derived from prepubertal boar testes were used as feeder layer and, when co-cultured with human sperm *in vitro*, were able to preserve normal sperm viability, motility and normal mitochondrial function after 7 days of culture [148]. Interestingly, the mitotic “quiescence” of mature Sertoli cells doesn’t seem to be related to their terminal differentiation. Instead, mature Sertoli cells resemble arrested proliferating cells: it was shown that the mature Sertoli cells from adult mouse and human testes were able to resume proliferation *in vitro* using standard culture conditions with no additives such as hormones or gonadotropins [149, 150].

Another strong (albeit indirect) argument in favor of Sertoli/SSCs cotransplantation potency comes from studies [151, 152]. In these studies, male germ cells from infertile Steel (*Sl/Sld*) mutant mice (in which the testes contain spermatogonia, but spermatogenesis does not occur due to the mutation-mediated Sertoli cell dysfunction) were transplanted into the infertile dominant white spotting (*W*) mutant male mice (with mutation-mediated germ cells dysfunction, but with preserved Sertoli cell function) and the transplantation resulted in donor-derived offspring. Evidently, the non-permissive testicular environment does not support functional spermatogonial stem cells properly and this can lead to animal infertility. Thus, the transplantation of only SSCs might be inefficient to restore fertility in recipients after gonadal ablation.

Several authors reported the Sertoli/SSCs cotransplantation [126, 153]. In these reports, the donor-derived “testicular cell suspension” (which included both, Sertoli cells and SSCs) was transplanted into gonadal ablated recipients. However, it is difficult to evaluate the role Sertoli cells play in the establishing of donor-derived spermatogenesis from such reports. On the other hand, to the best of our knowledge, no any study reported the cotransplantation of Sertoli cells with *in vitro* expanded and genome edited SSCs in livestock. We believe that the approach of Sertoli cells cotransplantation with *in vitro* expanded and genome edited SSCs is of great interest and, therefore, should be thoroughly examined in the future.

#### Mesenchymal or Leydig stem cells/SSCs cotransplantation

Gong et al. reported several lines of evidence (these including immunostaining and analysis of cytokines secretion) that rat Sertoli cells are, in fact, a kind of differentiated mesenchymal stem cells (MSCs) [154]. Moreover, it was shown, that Leydig cells also develop from undifferentiated mesenchymal-like stem cells [111], and that it is possible to differentiate MSC into testosterone-producing, Leydig-like cells with the use of several approaches [155, 156]. Therefore, the concept of MSC-SSC co-transplantation to increase colonization efficiency of donor-derived SSC by restoring the SSC niche after gonadal ablation in recipients is worth mentioning. Indeed, the improvements in SSC transplantation efficiency after MSC-SSC co-transplantation has recently been demonstrated in the mouse model [157].

Leydig stem cells could also be isolated based on their specific surface (CD51^+^) or intracellular (Nestin^+^) phenotypic markers with the use of immunostaining approach, as it was shown in mice [158]. *in vitro*, these self-renewal cells were capable of extensive proliferation and differentiation into mesenchymal or Leydig cells. *in vivo*, these cells were capable of differentiation into mature Leydig cells, and the recipient animals (rats) showed a partial recovery of testosterone production and spermatogenesis [158]. Therefore, the concept of Leydig stem cells/SSCs contransplantation to restore impaired testosterone secretion after testicular irradiation could also be valuable.

### Elimination of recipient’s endogenous germ cells via spermatogonia-specific approaches

An interesting and very promising approach of how to precisely eliminate the recipient’s endogenous SSCs with minimal (if any) negative impact on the neighboring somatic support cells might be the application of a gene editing tool to knockout (inactivate) one of the genes crucial for SSC development [159]. Recently, boars with the *NANOS2* gene knocked out were generated by a direct injection of the engineered CRISPR/Cas9 system into the cytoplasm of fertilized zygotes; this was followed by embryo transfer into estrus synchronized surrogate females [160]. The NANOS family of RNA binding proteins plays a crucial role in the development and maintenance of germline cells in males [160]. In boars, it was discovered that the inactivation of *NANOS2* leads to sperm loss in the ejaculate while preserving the intact seminiferous tubules and the functional testicular interstitial tissue [160]. Although the mechanisms of knockout-mediated germ cell loss have yet to be revealed, such genetically modified boars with ablated spermatogenesis might serve as ideal recipients for transplantation of the donor SSCs [160].

Recently, Herrid et al. reported selective toxicity of *Dolichos biflorus* agglutinin (DBA), a plant lectin, to spermatogonia in the bull and the dromedary camel [161]. DBA binds specifically to terminal N-acetylgalactosamine residues and was widely used for enrichment or labeling gonocytes or Type A spermatogonia in several livestock species, including bull, boar and stallion (reviewed in [161]). In the dromedary camel, a single dose of 25–50 μg/mL DBA injected into rete testis was shown to deplete endogenous stem cells in recipient testes [161]. Therefore, it was concluded, that DBA could be used effectively to eliminate endogenous stem cells.

As discussed by Smith et al. [162], other spermatogonia-specific approaches are available to eliminate the recipient’s endogenous germ cells in a controllable manner. For example, several transgenic approaches were reported in mice studies, which based on the use of the inhibin-alpha promoter/herpes simplex virus thymidine kinase transgene [163], the diphtheria toxin A chain gene directed by the histone H1t promoter [164] or the transgenic expression of an inducible primate iDTR within mouse germ cells [165].

### The ultrasonographic-guided SSC transplantation

**Fig. 4:**
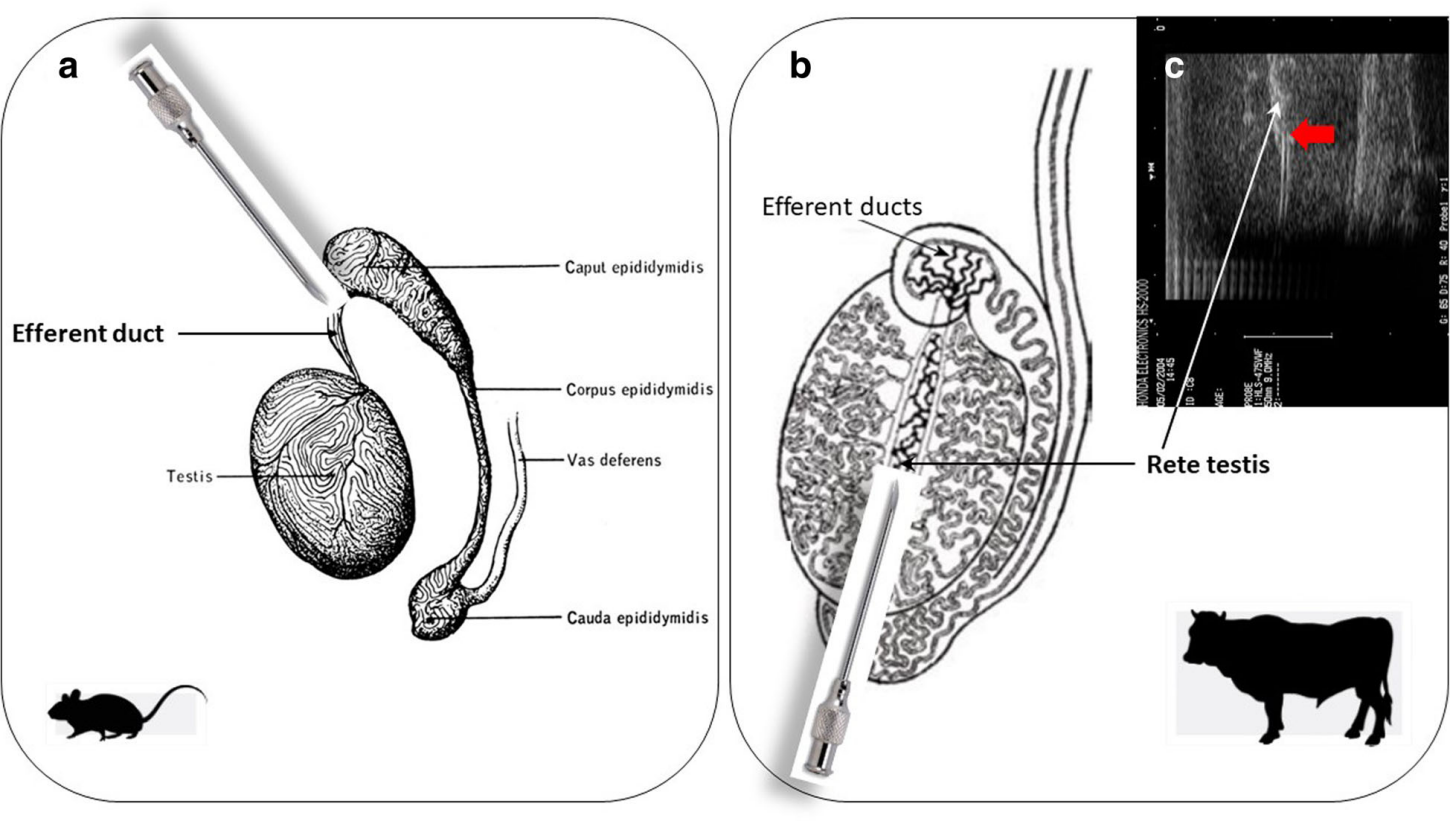
Schematic representation of the ultrasonographic-guided SSC transplantation technique. **a**. SSC transplantation in mouse. In this specie, there is a single efferent duct emerging from the rete testis, which is easy to cannulate to inject SSC. **b**. The ultrasonographic-guided cannulation of the centrally located rete testis in non-rodent animals. In livestock species, several efferent ducts emerge from the testis. That is why the transplantation of SSC is preferably done by cannulation of SSC into the rete testis with ultrasonographic guidance. **c**. The ultrasonographic guidance; rete testis is shown by the white arrow. Catheter, injected into the rete testis, is shown by the red arrow. Adapted from [52], modified. The ultrasonographic guidance picture is reproduced with a permission from the personal archive of professor Jonathan Hill, PhD

Briefly, in the SSC transplantation procedure [additional file 6], [additional file 7] , the SSCs are isolated from the testes of the donor animals and are transplanted by injection into the testicular seminiferous tubules of prepared recipients, where they initiate normal donor-derived spermatogenesis and result in functional sperm of donor-origin. A technique for SSC transplantation was initially developed using a mouse model in 1994 by Brinster and colleagues [166, 167]. Today, the SSC transplantation technique has been reported to be successful in several livestock species, including boar [121], ram [41], buck [123], bull [127] and dromedary camel [161]. However, this required the development of a new technical approach, the ultrasonographic-guided cannulation of the centrally located rete testis. This is because the direct injection of donor cells into seminiferous tubules is not possible via the efferent ducts in large species due to anatomic limitations. Ultrasound-guided cannulation could be completed in 15–30 min under general anesthesia and aseptic surgical conditions [82]. To enable successful transplantation, tens of millions of donor SSCs in the total volume of several mL would be enough to be injected per testis in large animals [49]. Ultrasound-guided cannulation is usually conducted under a flow rate of approximately 0.5–1 mL/min [82] and results in filling approximately half of the recipient’s seminiferous tubules with donor cells [51] (Fig. 4).

### Confirmation of transplanted donor SSC viability

Kim et al. [168] was able to obtain morphologically and functionally normal spermatozoa of donor origin after the SSC transplantation procedure in boars. Zeng et al. reported the production of transgenic donor-origin sperm after SSC transplantation in bucks [49]. Li et al. [57] demonstrated the self-renewal and spermatogenesis abilities of transplanted tree shrew SSCs, where donor-derived spermatogenesis persisted in recipients at day 250 post-transplantation. Recently, Herrid et al. [161] demonstrated for the first time, that the recipients produce spermatozoa of donor origin after heterologous testicular germ cell transplantation in dromedary camels. In all four studies, the donor origin was confirmed by the positive donor-origin gene expression of spermatozoa by polymerase chain reaction. It is important to mention that in certain livestock species, the transplanted transgenic SSCs will home into the niche, proliferate and produce an amount of transgenic sperm that will be detectable by PCR only after a certain duration of time (as long as 11 months in boars, [82]). The confirmation of the transplanted donor SSC viability by the production of donor-derived offspring (or transgenic IVF embryos) was described in recipient rams [169], bucks [87], boars [82] and cockerels [170]. Recent report of donor-origin sperm ejaculated by recipient in dromedary camel [161] indicated offspring may soon be produced also in this specie. In general, the transgene was stably integrated into the genome of the recipient animals, as it was possible to detect the transgene in ejaculates from recipient boars [82] or rams [169] at least 5 years after the transplant.

### SSC transplantation to extend the techniques of spermatozoa cryopreservation and/or artificial insemination

Sperm cryopreservation protocols (an integral component of the artificial insemination technique), if developed for individual livestock species, are often complicated, time-consuming, not interchangeable among species or not standardized. For example, due to high viscosity and poor quality of dromedary camel semen and due to unavailability of the standard protocol for diluting and freezing camel sperm, artificial insemination is not regularly used in camel breeding programs (discussed in [161]).

In contrast, SSC isolated from various livestock species might be successfully cryopreserved for a long period of time in liquid nitrogen, using invariable and well-established protocol, as is commonly done in cryopreservation of somatic cells [171]. It is important to mention the study of Redden et al. [172] who investigated the effectiveness of cryopreservation of bull testicular cells with different packaging procedures (large-sized cryostraws, cryobags or cryovials). The study confirmed that cryopreservation of testicular germ cells in 5 mL cryostraws at a density of up to 18 × 10^6^ cells/mL in liquid nitrogen appears to be a simple and practical way to preserve cells.

Further, it is known from the semen preservation industry that certain sires (so-called “bad freezers”) respond poorly to conventional freezing protocols [173]. This poor response in such sires could be partially improved if the conventional freezing protocol is modified [174]. Therefore, it was accepted that for the “bad freezers”, the conventional sperm freezing protocol is simply less-than-optimal and that the “bad freezers” produce sperm with a narrow tolerance to less-than-optimal conditions [174]. The “bad freezers” issue could be explained in terms of the concept of the spermatozoa heterogeneity (here the heterogeneity is the phenomenon of functional variability in the responses of spermatozoa, which is treated the same way [173]). A central idea behind this concept is that the spermatozoa in an individual’s ejaculate are very heterogeneous for several attributes, including their tolerance to cold-shock [173] and, importantly, their fertilizing potential [175]. The ejaculation of millions of sperm, all at a similar state of fertilizing potential, might suffice only for induced ovulators, such as cats or rabbits [175]. In contrast, it is especially important to consider the sperm heterogeneity in species with variable intervals between mating and ovulation, such as sheep [176]. Sperm become heterogeneous during their epididymal transit [173], and the subpopulations of spermatozoa with different fertilizing potentials arise [176].

Using techniques assessing sperm heterogeneity (such as the centrifugal countercurrent distribution technique), it was shown that ejaculate heterogeneity is positively correlated with sire field fertility [177]. Furthermore, the reduced fertilizing ability of the stored sperm was shown to be due to dramatic loss of sperm heterogeneity during cryopreservation because cooling, freezing and thawing produce homogeneous sperm samples with very limited functional versatility [173, 178]. Therefore, cryopreserved sperm represents a very limited genetic resource: the genetic variation is limited by the spermatozoa pool, which is derived from the thawed sperm sample.

Although it was reported that the cryopreservation is harmful also for germ cells and that cryosurvival rate of the preserved germline cells can be as low as 50% [179], cryopreserved SSC [additional file 8] are able to self-renew *in vitro* unlimitedly after thawing, thus providing the source of virtually any individual sire’s genetic program with full genetic variability within that program [171]. It has also been shown in mice studies that cryopreserved SSCs retain their spermatogenic function [171] and were able to successfully produce normal donor-derived offspring after transplantationing into testes of busulphan-sterilized recipient mice [180].

Furthermore, it is broadly accepted that SSC transplantation can be used for the propagation of elite sire genetics in extensive grazing systems (for instance, in beef cattle production), where the use of artificial insemination (AI) requires exhaustive management and is limited by complications related to, for example, estrous synchronization. Indeed, it was estimated that only approximately 10% of beef cows in the United States are bred using AI [181]. This means a lost opportunity for the genetic gain of beef cattle populations. On the other hand, the transplantation of SSCs between bulls would provide a tool (alternative to AI) for expanding the use of the genetic value of elite sires, and this will overcome the limitations of implementing an AI program [68].

## Conclusion

Apart from its value in extending the techniques of spermatozoa cryopreservation and/or artificial insemination, the immense promise of SSC manipulation lies in the acceleration of transgenesis in farm animals. In present review we covered several topics closely related to advances in the isolation and purification of livestock SSCs with such techniques as FACS, MACS, Staput velocity sedimentation or the differential plating technique. We reviewed advances in the establishment of effective long-term culture systems for the *in vitro* expansion of livestock germline stem cells. Furthermore, we reviewed advances in the precise genome editing of livestock MGSCs (especially with the use of CRISPR/Cas9, the most modern gene editing technology).

Based on the above reasons and the confirmed success of the SSC transplantation technique in bulls, boars, rams, bucks and dromedary camel, we conclude that the manipulation of spermatogonial stem cells is currently a feasible and affordable strategy for the genetic modification of livestock.

### Future directions

In previous years, several quite important PM- or SCNT-based approaches to generate transgenic farm animals via genomic editing by engineered nucleases in somatic cells were published: 1) the insertion of the mouse *SP110* gene into the genome of bovine macrophages to generate transgenic cattle with increased resistance to tuberculosis [86], 2) the knockout of the *PRNP* gene in immortalized bovine fibroblasts to generate prion-free cattle [182], 3) the disruption of the *CD163 subdomain 5* gene in porcine zygotes to generate pigs that are not susceptible to infection with porcine reproductive and respiratory syndrome virus [183], and 4) the targeting of the catalytic core of the PERV *pol* gene in primary porcine fetal fibroblasts to generate PERV-inactivated pigs [112]. In the future studies, we believe that (due to the reviewed disadvantages of the PM and SCNT methods), it is of great interest to revise or expand the abovementioned approaches using the CRISPR/Cas9 system in the context of livestock MGSC genomic editing.

## Additional files

Additional file 1: The choice of which donor population of germline stem cells to expand in culture is critical for the outcome of germ cell transplantation. In this sense, it is important to mention, that the use of PGCs is less practical as these cells are collected from embryo and there are just few PGCs per embryo [186, 187]. On the other hand, the SSCs (together with gonocytes) offer more practical option due to relatively simple procedure of their collection from the testes of neonates, juvenile or adult donors [12]. (DOCX 13 kb)

Additional file 2: Although not the focus of present review, it should be mentioned that this recent success with the establishment of stable bovine ES cell lines open the opportunity to revolutionize the livestock breeding. Using established pluripotent ES cells, germ cells can be induced *in vitro* to form functional spermatids and oocytes. Next, with the use of *in vitro* fertilization (IVF), embryos can be obtained from the *in vitro* generated spermatids and oocytes. Such an “animal embryo-stem cell breeding system” completes the whole livestock breeding scheme “in a dish” by integrating *in vitro* germ cell induction, IVF, genome sequencing, and genomic selection [188]. On the other hand, even the possibility of producing sperm *in vitro* would have had a great impact on livestock industries in case of success. As Aponte [52] has stated “…in the cattle industry, keeping animals in large facilities would be a thing of the past when renewable SSC pools from elite bulls produce high numbers of sperm in Petri dishes at small biotechnological facilities” (p.672). However, it is very important to take into consideration the possible effect of inbreeding after only using a limited number of available elite sires, and the consequent decrease of genetic variability in population [189]. (DOCX 12 kb)

Additional file 3: Because DSB are potentially lethal, the cell activates mechanisms to repair the DSB damage through the NHEJ or the HR processes, two major cellular DNA repair pathways [190]. The molecular nature of these pathways is complex, and a detailed overview of these pathways is outside the scope of the present review. Readers interesting in DNA repair by NHEJ or HR should refer reviews published elsewhere [190, 191]. However, for present review, it is important to introduce the difference between two: NHEJ is the more frequent, although imperfect, error-prone repair pathway that results in insertions and deletions (indels) at the break site [75]. These short DNA indels create targeted gene knockouts by inducing a frameshift of the amino acid codons and the formation of a premature stop codon [192]. On the other hand, HR is known to be more precise and is able to introduce the specific exogeneous nucleotide sequences into the repaired DNA (if donor template DNA is provided) [94]. (DOCX 12 kb)

Additional file 4: This could indicate either that a) donor stem cells are able to compete successfully with endogenous stem cells for available niches or b) there are vacant niches in the testes of livestock species that can be occupied by transplanted donor cells (discussed in [39]). (DOCX 11 kb)

Additional file 5: It is important to mention the study of Anand et al., who discussed the restoration of spermatogenesis by testicular transplantation of donor-derived Sertoli cells into busulphan-treated recipient mice [140]. According to the authors, spermatogenesis in the recipient was restored from a pool of endogenous (recipient-derived) very small embryonic-like stem cells (VSELs). These cells survived gonadal ablation, proliferated and gave rise to spermatogonial cells, but were unable to differentiate because of a compromised niche. Therefore, it is critical to confirm thoroughly the donor-origin of restored spermatogenesis after Sertoli cells co-transplantation. (DOCX 12 kb)

Additional file 6: In contrast to human research, intratesticular allo- (the transplantation between the different individuals of the same specie), or the xenotransplantation (the transplantation between individuals from different species) is mainly considered in livestock. (DOCX 13 kb)

Additional file 7: Alternativelly, ectopic transplantation of small (1–2 mm^3^) fragments of the testicular tissue isolated from livestock donor animal (the so-called xenografting approach) or of disassociated testicular cell suspension (the so-called *de novo* morphogenesis approach) under the dorsal skin of immunocompromised recipient mice could also be used to obtain fully functional haploid donor-derived spermatozoa [193, 194]. The capability of ectopically transplanted Sertoli cells to rearrange into seminiferous tubule-like structures to support donor-derived ectopic spermatogenesis is fascinating and is the fundamental of the *de novo* morphogenesis approach (discussed in [195]). Because of the use of mice models, both the xenografting and the *de novo* morphogenesis approaches help to overcome the costly and time-consuming process of maintaining large animal models in research. On the other hand, the practical application of both approaches in livestock breeding is notably limited by the needs to use the elaborative and costly techniques of assisted reproduction (such as intracytoplasmic sperm injection, ICSI) to generate the progeny from the obtained donor-derived spermatozoa. Therefore, both approaches are considered as invaluable *in vivo* bio-assays to comprehend spermatogenesis, however with low practical merit as of today. This is in contrast to SSCs intratesticular transplantation, which has its certain disadvantages if exploited as the experimental *in vivo* bio-assay but suits better to practical application in livestock breeding. Readers interested in the testicular tissue xenografting or *de novo* testicular morphogenesis should refer to the excellent reviews published elsewere [195, 196] or to several original papers, which confirm the exclusive experimental merit of these approaches in livestock research [197, 195]. (DOCX 13 kb)

Additional file 8: Compared to cryopreservation of single cell suspension, the approach of whole testis tissue cryopreservation is more challenging; this is because tissue requires longer exposure to cryoprotectants and this can result in higher cellular toxicity before freezing [135]. However, the cryopreservation of the whole testis tissue is recognized as promising, at least in human regenerative medicine. In this approach, the SSCs purified from the thawed tissue and propagated subsequently *in vitro* [198]. Readers interested in this approach should refer the very recent review [199]. (DOCX 12 kb)

## Abbreviations

AAV: Adeno-associated virus
AI: Artificial insemination
bFGF: Basic fibroblast growth factor
BSA: Bovine serum albumin
Cas9: CRISPR-associated protein 9, nuclease
Cas9-D10A: Variant of Cas9
Cas9n: Variant of Cas9
CD51: Cluster of designation, 51
CDH1: E-cadherin
Cpf1: Nuclease name, CRISPR from *Prevotella* and *Francisella* 1
CRISPR: Clustered regularly interspaced short palindromic repeats
crRNA: CRISPR RNA
CSF1: Colony-stimulating factor-1
DAF: Decay-accelerating factor
DAZL: Deleted in azoospermia-like
DBA: *Dolichos biflorus* agglutinin
DDX4: DEAD-box polypeptide 4
DSB: Double-stranded breaks
EDTA: Ethylenediaminetetraacetic acid
ES: Embryonic stem cells
FACS: Fluorescence-activated cell sorting
*Fok*I: Endonuclease, naturally found in *Flavobacterium okeanokoites*
GDNF: Glial cell line-derived neurotrophic factor
GFP: Green fluorescence protein
GFRα1: GDNF family receptor alpha-1
gRNA: Guide RNA
Gy: Grey, the irradiation unit
HR: Homologous recombination
ICSI: Intracytoplasmic sperm injection
iDTR: Mice line name, carrying a Cre-inducible simian diphtheria toxin receptor
ITGA6: Integrin alpha-6
IVF: *in vitro* fertilization
LE: Interstitial lymphatic endothelial cells
LV: Lentivirus-based vector
MACS: Magnetic activated cell sorting
MGSCs: Male germline stem cells
MSCs: Mesenchymal stem cells
NANOS2: the *Nanos* gene paralog. The NANOS family of RNA binding proteins play an essential role in the development of germline
NHEJ: Nonhomologous end-joining
PCR: Polymerase chain reaction
PERV: Porcine endogenous retrovirus
PGCs: Primordial germ cells
PGP9.5: Protein gene product 9.5
PLZF: Promyelocytic leukemia zinc finger protein
PM: Pronuclear microinjection
PMCs: Peritubular myoid cells
*pol*: (DNA polymerase) refers to a gene for reverse transcriptase in retroviruses. This enzyme transcribes the viral RNA into double-stranded DNA
*PRNP*: The gene provides instructions for making a protein called prion protein
SCNT: Somatic cell nuclear transfer
*SP110*: Speckled 110 KDa, the nuclear body protein gene. The product of the gene control the growth of *Mycobacterium tuberculosis* in macrophages and induce apoptosis in infected cells
SSCs: Spermatogonial stem cells
TALEN: Transcription activator-like effector nucleases
tracrRNA: Trans-activating crRNA
VEGF: Vascular endothelial growth factor
VSELs: Very small embryonic-like stem cells
ZFN: Zinc-finger nucleases
