# Supplementary material for: Manipulation of spermatogonial stem cells in livestock species
Source: J Anim Sci Biotechnol. 2019 Jun 12;10:46. doi: 10.1186/s40104-019-0355-4 (PMC6560896; doi:10.1186/s40104-019-0355-4)
Supplement: Supplementary file 1 — The choice of which donor population of germline stem cells to expand in culture is critical for the outcome of germ cell transplantation. In this sense, it is important to mention, that the use of PGCs is less practical as these cells are collected from embryo and there are just few PGCs per embryo [186, 187]. On the other hand, the SSCs (together with gonocytes) offer more practical option due to relatively simple procedure of their collection from the testes of neonates, juvenile or adult donors [12]. (DOCX 13 kb) [file 40104_2019_355_MOESM1_ESM.docx]

[additional file 1] The choice of which donor population of germline stem cells to expand in culture is critical for the outcome of germ cell transplantation. In this sense, it is important to mention, that the use of PGCs is less practical as these cells are collected from embryo and there are just few PGCs per embryo [186, 187]. On the other hand, the SSCs (together with gonocytes) offer more practical option due to relatively simple procedure of their collection from the testes of neonates, juvenile or adult donors [12].

**References for the additional file 1**

186. Jiang FX, Short RV. Different fate of primordial germ cells and gonocytes following transplantation. APMIS. 1998; doi: 10.1111/j.1699-0463.1998.tb01319.x

187. Ohta H, Wakayama T, Nishimune Y. Commitment of fetal male germ cells to spermatogonial stem cells during mouse embryonic development. Biol Reprod. 2004; doi: 10.1095/biolreprod.103.024612
