# Supplementary material for: Manipulation of spermatogonial stem cells in livestock species
Source: J Anim Sci Biotechnol. 2019 Jun 12;10:46. doi: 10.1186/s40104-019-0355-4 (PMC6560896; doi:10.1186/s40104-019-0355-4)
Supplement: Supplementary file 2 — Although not the focus of present review, it should be mentioned that this recent success with the establishment of stable bovine ES cell lines open the opportunity to revolutionize the livestock breeding. Using established pluripotent ES cells, germ cells can be induced in vitro to form functional spermatids and oocytes. Next, with the use of in vitro fertilization (IVF), embryos can be obtained from the in vitro generated spermatids and oocytes. Such an “animal embryo-stem cell breeding system” completes the whole livestock breeding scheme “in a dish” by integrating in vitro germ cell induction, IVF, genome sequencing, and genomic selection [188]. On the other hand, even the possibility of producing sperm in vitro would have had a great impact on livestock industries in case of success. As Aponte [52] has stated “…in the cattle industry, keeping animals in large facilities would be a thing of the past when renewable SSC pools from elite bulls produce high numbers of sperm in Petri dishes at small biotechnological facilities” (p.672). However, it is very important to take into consideration the possible effect of inbreeding after only using a limited number of available elite sires, and the consequent decrease of genetic variability in population [189]. (DOCX 12 kb) [file 40104_2019_355_MOESM2_ESM.docx]

[additional file 2] Although not the focus of present review, it should be mentioned that this recent success with the establishment of stable bovine ES cell lines open the opportunity to revolutionize the livestock breeding. Using established pluripotent ES cells, germ cells can be induced *in vitro* to form functional spermatids and oocytes. Next, with the use of *in vitro* fertilization (IVF), embryos can be obtained from the *in vitro* generated spermatids and oocytes. Such an “animal embryo-stem cell breeding system” completes the whole livestock breeding scheme “in a dish” by integrating *in vitro* germ cell induction, IVF, genome sequencing, and genomic selection [188]. On the other hand, even the possibility of producing sperm *in vitro* would have had a great impact on livestock industries in case of success. As Aponte [52] has stated “…in the cattle industry, keeping animals in large facilities would be a thing of the past when renewable SSC pools from elite bulls produce high numbers of sperm in Petri dishes at small biotechnological facilities” (p.672). However, it is very important to take into consideration the possible effect of inbreeding after only using a limited number of available elite sires, and the consequent decrease of genetic variability in population [189].

**References for the additional file 2**

188. Hou Z, An L, Han J, Yuan Y, Chen D, Tian J. Revolutionize livestock breeding in the future: an animal embryo-stem cell breeding system in a dish. J Anim Sci Biotechnol. 2018; doi: 10.1186/s40104-018-0304-7

189. Ptáček M, Ducháček J, Schmidová J, Stádník L. Response to selection of a breeding program for Suffolk sheep in the Czech Republic. Czech J. Anim. Sci. 2018; doi: 10.17221/21/2018-CJAS
