# Supplementary material for: Manipulation of spermatogonial stem cells in livestock species
Source: J Anim Sci Biotechnol. 2019 Jun 12;10:46. doi: 10.1186/s40104-019-0355-4 (PMC6560896; doi:10.1186/s40104-019-0355-4)
Supplement: Supplementary file 3 — Because DSB are potentially lethal, the cell activates mechanisms to repair the DSB damage through the NHEJ or the HR processes, two major cellular DNA repair pathways [190]. The molecular nature of these pathways is complex, and a detailed overview of these pathways is outside the scope of the present review. Readers interesting in DNA repair by NHEJ or HR should refer reviews published elsewhere [190, 191]. However, for present review, it is important to introduce the difference between two: NHEJ is the more frequent, although imperfect, error-prone repair pathway that results in insertions and deletions (indels) at the break site [75]. These short DNA indels create targeted gene knockouts by inducing a frameshift of the amino acid codons and the formation of a premature stop codon [192]. On the other hand, HR is known to be more precise and is able to introduce the specific exogeneous nucleotide sequences into the repaired DNA (if donor template DNA is provided) [94]. (DOCX 12 kb) [file 40104_2019_355_MOESM3_ESM.docx]

[additional file 3] Because DSB are potentially lethal, the cell activates mechanisms to repair the DSB damage through the NHEJ or the HR processes, two major cellular DNA repair pathways [190]. The molecular nature of these pathways is complex, and a detailed overview of these pathways is outside the scope of the present review. Readers interesting in DNA repair by NHEJ or HR should refer reviews published elsewhere [190, 191]. However, for present review, it is important to introduce the difference between two: NHEJ is the more frequent, although imperfect, error-prone repair pathway that results in insertions and deletions (indels) at the break site [75]. These short DNA indels create targeted gene knockouts by inducing a frameshift of the amino acid codons and the formation of a premature stop codon [192]. On the other hand, HR is known to be more precise and is able to introduce the specific exogeneous nucleotide sequences into the repaired DNA (if donor template DNA is provided) [94].

**References for the additional file 3**

190. Chapman JR, Taylor MR, Boulton SJ. Playing the end game: DNA double-strand break repair pathway choice. Mol Cell. 2012; doi: 10.1016/j.molcel.2012.07.029

191. Carroll D, Beumer KJ. Genome Engineering with TALENs and ZFNs: Repair pathways and donor design. Methods. 2014; doi: 10.1016/j.ymeth.2014.03.026

192. Christian M, Cermak T, Doyle EL, Schmidt C, Zhang F, Hummel A, et al. Targeting DNA double-strand breaks with TAL effector nucleases. Genetics. 2010; doi: 10.1534/genetics.110.120717
