# Supplementary material for: Manipulation of spermatogonial stem cells in livestock species
Source: J Anim Sci Biotechnol. 2019 Jun 12;10:46. doi: 10.1186/s40104-019-0355-4 (PMC6560896; doi:10.1186/s40104-019-0355-4)
Supplement: Supplementary file 4 — This could indicate either that a) donor stem cells are able to compete successfully with endogenous stem cells for available niches or b) there are vacant niches in the testes of livestock species that can be occupied by transplanted donor cells (discussed in [39]). (DOCX 11 kb) [file 40104_2019_355_MOESM4_ESM.docx]

[additional file 4] This could indicate either that a) donor stem cells are able to compete successfully with endogenous stem cells for available niches or b) there are vacant niches in the testes of livestock species that can be occupied by transplanted donor cells (discussed in [39]).
