# Supplementary material for: Manipulation of spermatogonial stem cells in livestock species
Source: J Anim Sci Biotechnol. 2019 Jun 12;10:46. doi: 10.1186/s40104-019-0355-4 (PMC6560896; doi:10.1186/s40104-019-0355-4)
Supplement: Supplementary file 5 — It is important to mention the study of Anand et al., who discussed the restoration of spermatogenesis by testicular transplantation of donor-derived Sertoli cells into busulphan-treated recipient mice [140]. According to the authors, spermatogenesis in the recipient was restored from a pool of endogenous (recipient-derived) very small embryonic-like stem cells (VSELs). These cells survived gonadal ablation, proliferated and gave rise to spermatogonial cells, but were unable to differentiate because of a compromised niche. Therefore, it is critical to confirm thoroughly the donor-origin of restored spermatogenesis after Sertoli cells co-transplantation. (DOCX 12 kb) [file 40104_2019_355_MOESM5_ESM.docx]

[additional file 5] It is important to mention the study of Anand et al., who discussed the restoration of spermatogenesis by testicular transplantation of donor-derived Sertoli cells into busulphan-treated recipient mice [140]. According to the authors, spermatogenesis in the recipient was restored from a pool of endogenous (recipient-derived) very small embryonic-like stem cells (VSELs). These cells survived gonadal ablation, proliferated and gave rise to spermatogonial cells, but were unable to differentiate because of a compromised niche. Therefore, it is critical to confirm thoroughly the donor-origin of restored spermatogenesis after Sertoli cells co-transplantation.
