# Supplementary material for: Manipulation of spermatogonial stem cells in livestock species
Source: J Anim Sci Biotechnol. 2019 Jun 12;10:46. doi: 10.1186/s40104-019-0355-4 (PMC6560896; doi:10.1186/s40104-019-0355-4)
Supplement: Supplementary file 6 — In contrast to human research, intratesticular allo- (the transplantation between the different individuals of the same specie), or the xenotransplantation (the transplantation between individuals from different species) is mainly considered in livestock. (DOCX 13 kb) [file 40104_2019_355_MOESM6_ESM.docx]

[additional file 6] In contrast to human research, intratesticular allo- (the transplantation between the different individuals of the same specie), or the xenotransplantation (the transplantation between individuals from different species) is mainly considered in livestock.
