# Supplementary material for: Manipulation of spermatogonial stem cells in livestock species
Source: J Anim Sci Biotechnol. 2019 Jun 12;10:46. doi: 10.1186/s40104-019-0355-4 (PMC6560896; doi:10.1186/s40104-019-0355-4)
Supplement: Supplementary file 7 — Alternativelly, ectopic transplantation of small (1–2 mm3) fragments of the testicular tissue isolated from livestock donor animal (the so-called xenografting approach) or of disassociated testicular cell suspension (the so-called de novo morphogenesis approach) under the dorsal skin of immunocompromised recipient mice could also be used to obtain fully functional haploid donor-derived spermatozoa [193, 194]. The capability of ectopically transplanted Sertoli cells to rearrange into seminiferous tubule-like structures to support donor-derived ectopic spermatogenesis is fascinating and is the fundamental of the de novo morphogenesis approach (discussed in [195]). Because of the use of mice models, both the xenografting and the de novo morphogenesis approaches help to overcome the costly and time-consuming process of maintaining large animal models in research. On the other hand, the practical application of both approaches in livestock breeding is notably limited by the needs to use the elaborative and costly techniques of assisted reproduction (such as intracytoplasmic sperm injection, ICSI) to generate the progeny from the obtained donor-derived spermatozoa. Therefore, both approaches are considered as invaluable in vivo bio-assays to comprehend spermatogenesis, however with low practical merit as of today. This is in contrast to SSCs intratesticular transplantation, which has its certain disadvantages if exploited as the experimental in vivo bio-assay but suits better to practical application in livestock breeding. Readers interested in the testicular tissue xenografting or de novo testicular morphogenesis should refer to the excellent reviews published elsewere [195, 196] or to several original papers, which confirm the exclusive experimental merit of these approaches in livestock research [197, 195]. (DOCX 13 kb) [file 40104_2019_355_MOESM7_ESM.docx]

[additional file 7] Alternativelly, ectopic transplantation of small (1 - 2 mm^3^) fragments of the testicular tissue isolated from livestock donor animal (the so-called xenografting approach) or of disassociated testicular cell suspension (the so-called *de novo* morphogenesis approach) under the dorsal skin of immunocompromised recipient mice could also be used to obtain fully functional haploid donor-derived spermatozoa [193, 194]. The capability of ectopically transplanted Sertoli cells to rearrange into seminiferous tubule-like structures to support donor-derived ectopic spermatogenesis is fascinating and is the fundamental of the *de novo* morphogenesis approach (discussed in [195]). Because of the use of mice models, both the xenografting and the *de novo* morphogenesis approaches help to overcome the costly and time-consuming process of maintaining large animal models in research. On the other hand, the practical application of both approaches in livestock breeding is notably limited by the needs to use the elaborative and costly techniques of assisted reproduction (such as intracytoplasmic sperm injection, ICSI) to generate the progeny from the obtained donor-derived spermatozoa. Therefore, both approaches are considered as invaluable *in vivo* bio-assays to comprehend spermatogenesis, however with low practical merit as of today. This is in contrast to SSCs intratesticular transplantation, which has its certain disadvantages if exploited as the experimental *in vivo* bio-assay but suits better to practical application in livestock breeding. Readers interested in the testicular tissue xenografting or *de novo* testicular morphogenesis should refer to the excellent reviews published elsewere [195, 196] or to several original papers, which confirm the exclusive experimental merit of these approaches in livestock research [197, 195].

**References for the additional file 7**

193. Nakai M, Kaneko H, Somfai T, Maedomari N, Ozawa M, Noguchi J, et al. Production of viable piglets for the first time using sperm derived from ectopic testicular xenografts. Reproduction. 2010; doi: 10.1530/REP-09-0509

194. Honaramooz A, Megee SO, Rathi R, Dobrinski I. Building a testis: formation of functional testis tissue after transplantation of isolated porcine (Sus scrofa) testis cells. Biol Reprod. 2007; Jan;76(1):43-7

195. Dores C, Alpaugh W, Dobrinski I. From in vitro culture to in vivo models to study testis development and spermatogenesis. Cell Tissue Res. 2012; doi: 10.1007/s00441-012-1457-x

196. Arregui L, Dobrinski I. Xenografting of testicular tissue pieces: 12 years of an in vivo spermatogenesis system. Reproduction. 2014; doi: 10.1530/REP-14-0249

197. Kaneko H, Kikuchi K, Nakai M, Fuchimoto D, Suzuki S, Sembon S, et al. Establishment of a strain of haemophilia-A pigs by xenografting of foetal testicular tissue from neonatally moribund cloned pigs. Sci Rep. 2017; doi: 10.1038/s41598-017-17017-6
