# Supplementary material for: Manipulation of spermatogonial stem cells in livestock species
Source: J Anim Sci Biotechnol. 2019 Jun 12;10:46. doi: 10.1186/s40104-019-0355-4 (PMC6560896; doi:10.1186/s40104-019-0355-4)
Supplement: Supplementary file 8 — Compared to cryopreservation of single cell suspension, the approach of whole testis tissue cryopreservation is more challenging; this is because tissue requires longer exposure to cryoprotectants and this can result in higher cellular toxicity before freezing [135]. However, the cryopreservation of the whole testis tissue is recognized as promising, at least in human regenerative medicine. In this approach, the SSCs purified from the thawed tissue and propagated subsequently in vitro [198]. Readers interested in this approach should refer the very recent review [199]. (DOCX 12 kb) [file 40104_2019_355_MOESM8_ESM.docx]

[additional file 8] Compared to cryopreservation of single cell suspension, the approach of whole testis tissue cryopreservation is more challenging; this is because tissue requires longer exposure to cryoprotectants and this can result in higher cellular toxicity before freezing [135]. However, the cryopreservation of the whole testis tissue is recognized as promising, at least in human regenerative medicine. In this approach, the SSCs purified from the thawed tissue and propagated subsequently *in vitro* [198]. Readers interested in this approach should refer the very recent review [199].

**References for the additional file 8**

198. Sadri-Ardekani H, McLean TW, Kogan S, Sirintrapun J, Crowell K, Yousif MQ, et al. Experimental testicular tissue banking to generate spermatogenesis in the future: A multidisciplinary team approach. Methods. 2016; doi: 10.1016/j.ymeth.2016.02.013

199. Zarandi NM, Galdon G, Kogan S, Atala A, Sadri-Ardekani H. Cryostorage of immature and mature human testis tissue to preserve spermatogonial stem cells (SSCs): a systematic review of current experiences toward clinical applications. Stem Cells Cloning. 2018; doi: 10.2147/SCCAA.S137873
